# Identification and characterisation of CYP75A31, a new flavonoid 3'5'-hydroxylase, isolated from *Solanum lycopersicum*

**DOI:** 10.1186/1471-2229-10-21

**Published:** 2010-02-03

**Authors:** Kristine M Olsen, Alain Hehn, Hélène Jugdé, Rune Slimestad, Romain Larbat, Frédéric Bourgaud, Cathrine Lillo

**Affiliations:** 1 University of Stavanger, Centre for Organelle Research, Faculty of Science and Technology, N-4036 Stavanger, Norway; 2 UMR 1121 Nancy Université (INPL)-INRA Agronomie et Environnement (Nancy/Colmar), IFR110, 2, av. de la Forêt de Haye, 54505 Vandoeuvre-lès-Nancy, France; 3 PlantChem, Saerheim Research Center, N-4353 Klepp stasjon, Norway

## Abstract

**Background:**

Understanding the regulation of the flavonoid pathway is important for maximising the nutritional value of crop plants and possibly enhancing their resistance towards pathogens. The flavonoid 3'5'-hydroxylase (F3'5'H) enzyme functions at an important branch point between flavonol and anthocyanin synthesis, as is evident from studies in petunia (*Petunia hybrida*), and potato (*Solanum tuberosum*). The present work involves the identification and characterisation of a *F3'5'H *gene from tomato (*Solanum lycopersicum*), and the examination of its putative role in flavonoid metabolism.

**Results:**

The cloned and sequenced tomato *F3'5'H *gene was named *CYP75A31*. The gene was inserted into the *pYeDP60 *expression vector and the corresponding protein produced in yeast for functional characterisation. Several putative substrates for F3'5'H were tested *in vitro *using enzyme assays on microsome preparations. The results showed that two hydroxylation steps occurred. Expression of the *CYP75A31 *gene was also tested *in vivo*, in various parts of the vegetative tomato plant, along with other key genes of the flavonoid pathway using real-time PCR. A clear response to nitrogen depletion was shown for *CYP75A31 *and all other genes tested. The content of rutin and kaempferol-3-rutinoside was found to increase as a response to nitrogen depletion in most parts of the plant, however the growth conditions used in this study did not lead to accumulation of anthocyanins.

**Conclusions:**

*CYP75A31 *(NCBI accession number GQ904194), encodes a flavonoid 3'5'-hydroxylase, which accepts flavones, flavanones, dihydroflavonols and flavonols as substrates. The expression of the *CYP75A31 *gene was found to increase in response to nitrogen deprivation, in accordance with other genes in the phenylpropanoid pathway, as expected for a gene involved in flavonoid metabolism.

## Background

Flavonoids are plant secondary metabolites. They have a wide range of functions such as (a) providing pigmentation to flowers, fruits, and seeds in order to attract pollinators and seed dispersers, (b) protecting against ultraviolet light, (c) providing defence against phytopathogens (pathogenic microorganisms, insects, animals), (d) playing a role in plant fertility and germination of pollen and (e) acting as signal molecules in plant-microbe interactions [1,2]. Flavonoids receive a lot of attention due to their possible effects on human health. Many flavonoids display antioxidant activity that confers beneficial effects on coronary heart disease, cancer, and allergies [3,4]. Reports also suggest that some of the biological effects of anthocyanins and flavonols may be related to their ability to modulate mammalian cell signalling pathways [5,6]. Enhancing the production of flavonoids in crop plants can therefore give an important boost to their nutritional value, which makes knowledge of expression and regulation of the flavonoid pathway important. Flavonoids constitute a relatively diverse family of aromatic molecules that are derived from phenylalanine and malonyl-coenzyme A. Most of the bright red and blue colours found in higher plants are due to anthocyanins. Anthocyanin biosynthesis has been studied extensively in several plant species and detailed information on the pathway is available [7-9]. Information on substrate flow and regulation through the branch point between flavonol and anthocyanin synthesis is however not fully elucidated, and for tomato the enzymes acting in the branch point have not been extensively characterised. Experiments with expression of the snapdragon transcription factor genes *Delila*, a basic-helix-loop-helix (bHLH) transcription factor, and *Rosea1*, a R2R3 MYB-type transcription factor, showed that *F3'5'H *expression is necessary for activation of anthocyanin synthesis in tomatoes [10]. Introduction of these transcription factors under control of the fruit-specific E8 promoter increased the expression of most of the structural genes in the biosynthetic pathway in the tomato fruit, including *phenylalanine ammonia-lyase *(*PAL*), *chalcone isomerase *(*CHI*) and *F3'5'H*. *PAL *insures high flux into the phenylpropanoid pathway, whereas *CHI *and *F3'5'H *are essential for addressing the flux towards flavonoids in general and anthocyanin production specifically. The activity of CHI is normally low in the tomato skin, leading to accumulation of naringenin-chalcone in the skin of wild type tomatoes [11]. The cytochrome P450 dependent flavonoid hydroxylases introduce either one (flavonoid 3'-hydroxylase, F3'H) or two (F3'5'H) of the hydroxyl groups on the B ring of the flavonoid skeleton [7,12]. The F3'5'H belongs to the CYP75 superfamily of P450 enzymes [13,14]. These enzymes are anchored to the surface of the endoplasmic reticulum via their hydrophobic N- terminal end. Only plants that express the *F3'5'H *gene are capable of producing blue flowers, as these are dependent on 5'-hydroxylated anthocyanins. F3'5'-hydroxylases are previously known from other plants, such as *Petunia hybrida *(petunia), *Catharanthus roseus *(Madagascar periwinkle), *Vitis vinifera *(grape), *Campanula medium *(Canterbury bells), *Solanum tuberosum *(potato) and *Solanum melongena *(eggplant), among others. To be active P450 enzymes need to be coupled to an electron donor. This can either be a cytochrome P450 reductase or cytochrome b_5_. The reductase will also be anchored to the surface of the endoplasmic reticulum via its N- or C-terminus [13]. Kaltenbach et al. [15] isolated the *F3'5'H *gene from *C. roseus *using heterologous screening with the CYP75 *Hf1 *cDNA from *P. hybrida *[16]. Both the *C. roseus *gene, named CYP75A8, and the petunia *Hf1 *were expressed in *E. coli *and found to accept flavones, flavanones, dihydroflavonols and flavonols as substrates, and both performed 3'- and 3'5'-hydroxylation. The genes encoding *F3'5'H *in grape have been shown to be expressed in different parts of the grape plant that accumulate flavonoids, especially in the skin of ripening berries where the highest levels of anthocyanins are synthesized [17].

**Figure 1:**
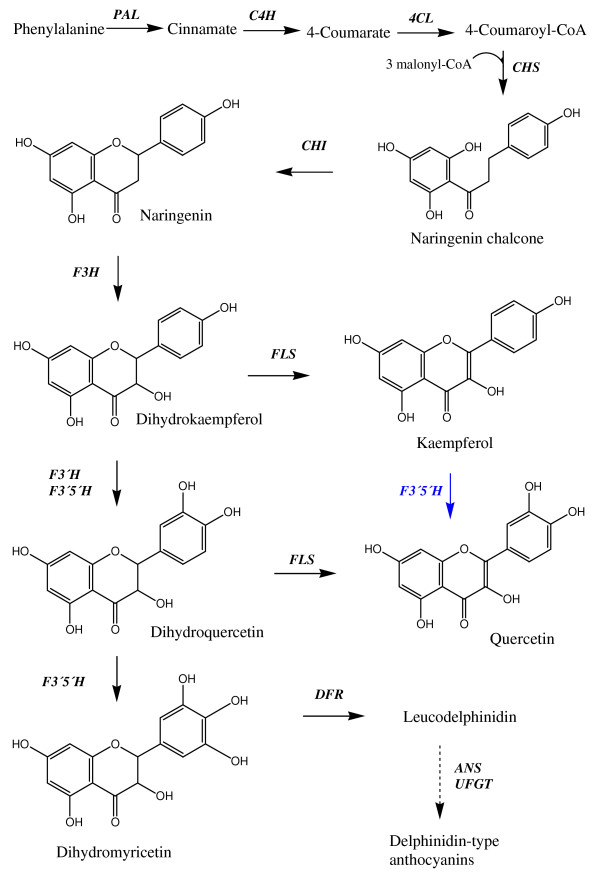
**Simplified scheme of the phenylpropanoid pathway in tomato**. The first committed enzyme in the flavonoid pathway is CHS. The reaction indicated in blue has been proven *in vitro *in this study, however it is unclear if it occurs *in planta*. Enzymes are given in bold italics. *PAL: phenylalanine ammonia-lyase. C4H: cinnamate 4-hydroxylase. 4CL: 4-coumarate: CoA ligase. CHS: chalcone synthase. CHI: chalcone isomerase. F3H: flavanone 3-hydroxylase. FLS: flavonol synthase. F3'H: flavonoid 3'-hydroxylase. F3'5'H: flavonoid 3'5'-hydroxylases. DFR: dihydroflavonol 4-reductase. ANS: anthocyanidin synthase. UFGT: UDP glucose flavonoid 3-O-glucosyl transferase*.

Several genes in the flavonoid pathway display differences in substrate specificity or preference in various plant species. Petunia dihydroflavonol 4-reductase (DFR), for instance, does not utilize dihydrokaempferol [18]. Arabidopsis DFR converts dihydroquercetin into leuco-cyanidin, but will use dihydrokaempferol when dihydroquercetin is not available, e.g. in plants lacing functional F3'H enzyme [19]. This is because the plants lacking F3'H activity cannot produce dihydroquercetin (fig 1). So far there is not much information on F3'5'H substrate specificity. Available data [15,20] generally confirm the same substrates, without reporting negative results for other substrates tested. However, Tanaka et al. [20] reported that the petunia *Hf2 *cDNA expressed in a yeast system did not accept apigenin as substrate. Kaltenbach et al. did, however, show that the petunia *Hf1 *can accept apigenin as substrate, when expressed in an *E. coli *system [15].

F3'5'H competes with flavonol synthase (FLS) for the substrates dihydrokaempferol and dihydroquercetin (Figure 1). The preferred substrate for DFR in the tomato plant is dihydromyricetin [21], which can be produced from dihydrokaempferol and dihydroquercetin by F3'5'H. This is the first step in the branch leading to anthocyanins (delphinidin type), which are normally only found in the vegetative tissues of tomato. According to Bovy et al. [21] tomato FLS prefers dihydroquercetin and dihydrokaempferol as substrates, and does not use dihydromyricetin, hence DFR and FLS do not compete for the same substrate. Nevertheless FLS can still deplete the flow of substrate towards DFR by using dihydrokaempferol and dihydroquercetin as they precede dihydromyricetin in the synthesis pathway. F3'H might also compete with FLS and F3'5'H for dihydrokaempferol, although it is unclear, as the enzyme has not been characterised from tomato so far. The activities of FLS, F3'5'H, DFR, and possibly F3'H, hence regulate the distribution between flavonols and anthocyanins in tomato plants. As a consequence, F3'5'H can be a bottleneck in this system as DFR relies on its activity to proceed the synthesis towards anthocyanins. Bovy et al. [11] has shown that silencing of the *FLS *gene leads to more anthocyanins in vegetative tomato tissue. Introduction of an *FLS *RNAi construct into tomato plants led to decreased levels of quercetin-3-rutinoside (rutin) in tomato peel, and to accumulation of anthocyanins in leaves, stems and flower buds. This indicates that less competition from flavonol synthesis will enhance the flux towards anthocyanins by allowing more substrate for DFR. In this study we cloned, sequenced and characterised the F3'5'H enzyme, which produces substrate for DFR in tomato. Accumulation of flavonoids, and distribution of products through the different branches of the flavonoid pathway, has previously been shown to be influenced by nitrogen supply [22,23]. An agricultural plant like tomato is typically given nitrogen through fertilization; hence the level of nitrogen available to the plant can be monitored. It is, therefore, important to elucidate the effects nitrogen has on expression of genes and accumulation of compounds, such as flavonoids. Extensive knowledge on the branch-point enzyme F3'5'H is crucial for understanding the distribution of flow through the flavonoid pathway, potentially enabling manipulation of desired end-product accumulation in fruits and vegetables in response to growth conditions.

## Results

### Sequence analysis

**Figure 2:**
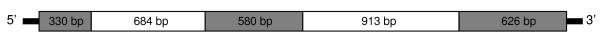
**Gene model of *CYP75A31***. The *CYP75A31 *gene isolated form the tomato cultivar Suzanne F1 consists of 3 exons (gray) and 2 introns. GenBank accession number: GQ904194.

The *CYP75A31 *gene was isolated using sequence homology with a potato *F3'5'H *and 3' RACE to identify the 3' end of the gene. A tomato EST sequence found in the TIGR database was assumed to be the 5' end of the gene (accession number DB723744), and primers based on these sequences led to isolation of the cDNA and DNA sequences for *CYP75A31*. The 3133 bp gene sequence (Figure 2) consists of three exons (gray), which is consistent with what is previously reported for potato, petunia and soybean [24,25]. A Blast search (NCBI) performed with the coding sequence revealed 94% identity to a *S. tuberosum*, 88% identity to a *S. melongena *and 84% identity to a *P. hybrida F3'5'H *sequence.

### Phylogenetic analysis

**Figure 3:**
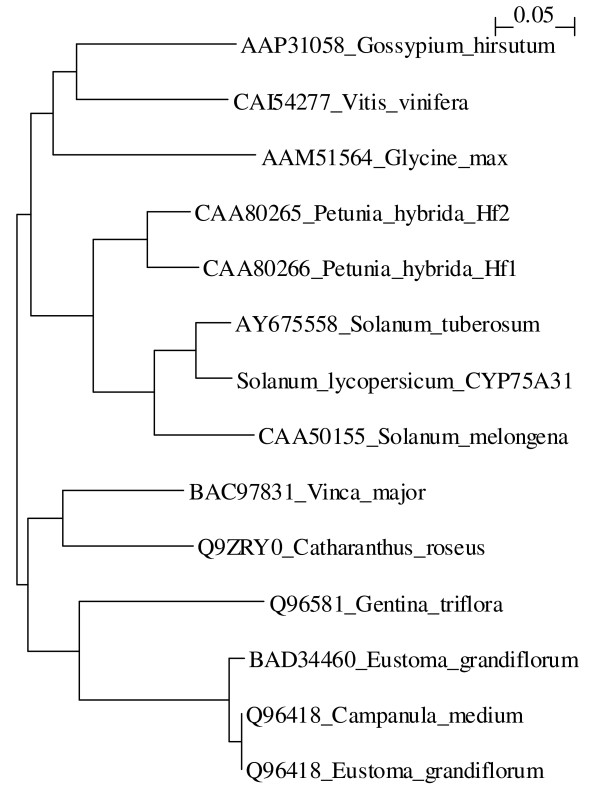
**Phylogenetic tree for a selection of F3'5'H enzymes**. The phylogenetic tree was made using protein sequences from several plant F3'5'H enzymes retrieved from the NCBI web page. Accession numbers are displayed in the figure.

The phylogenetic tree (Figure 3) was made using protein sequences from several plant F3'5'H enzymes retrieved from the NCBI web page. The tree clearly visualises that CYP75A31 is most closely related to the F3'5'H enzymes of the Solanum species potato and eggplant.

### CYP75A31 Substrate Specificity

The coding sequence of the *CYP75A31 *gene was transformed into yeast for heterologous expression. Enzyme assays were run on isolated microsome fractions, substrates and products were analysed by HPLC and MS. The substrates found to be metabolized by CYP75A31 are listed in table 1. Luteolin (5,7,3',4'-tetrahydroxyflavone) gave tricetin (5,7,3',4',5'-pentahydroxyflavone) as the only product. Naringenin (5,7,4'-trihydroxyflavanone) gave rice to two peaks in the HPLC-spectrum identified as eriodictyol (5,7,3',4'-tetrahydroxyflavanone), and 5,7,3',4',5'-pentahydroxyflavanone. As expected, eriodictyol as substrate gave only one product, 5,7,3',4',5'-pentahydroxyflavanone. Dihydrokaempferol (3,5,7,4'-tetrahydroxyflavanone) gave two peaks, dihydroquercetin (3,5,7,3',4'-pentahydroxyflavanone), and dihydromyricetin (3,5,7,3',4',5'-hexahydroxyflavanone). Dihydroquercetin as substrate gave one product, as expected, identified as dihydromyricetin. Kaempferol (3,5,7,4'-tetrahydroxyflavone) resulted in two peaks, identified as quercetin (3,5,7,3',4'-pentahydroxyflavone) and myricetin (3,5,7,3',4',5'-hexahydroxyflavone). Quercetin as substrate gave myricetin as the only product, and liquiritigenin (7,4'-dihydroxyflavanone) gave two products: butin (7,3',4'-trihydroxyflavanone) and 7,3',4',5'-tetrahydroxyflavanone. Neither the control reactions without NADPH, nor assays with microsomes isolated from yeast transformed with *pYeDP60 *vector lacking an insertion, showed any product formation.

**Table 1:** List of accepted substrates for CYP75A31

Substrate	Product of 3'-hydroxylation	Product of 5'-hydroxylation	Class
Luteolin (20.3) [286]	-	Tricetin (18.2) [302]	Flavone

Naringenin (21.2) [272]	Eriodictyol (19.1) [288]	5,7,3',4',5'-pentahydroxyflavanone (16.3)	Flavanone

Eriodictyol (18.9) [288]	-	5,7,3',4',5'-pentahydroxyflavanone (16.2) [304]	Flavanone

Dihydrokaempferol (17.0) [288]	Dihydroquercetin (15.0) [304]	Dihydromyricetin (12.4) [320]	Dihydroflavonol

Dihydroquercetin (15.0) [302]	-	Dihydromyricetin (12.4) [318]	Dihydroflavonol

Kaempferol (22.4) [286]	Quercetin (20.1) [302]	Myricetin (17.1) [318]	Flavonol

Quercetin (20.0) [302]	-	Myricetin (17.0) [318]	Flavonol

Liquiritigenin (19.0) [256]	Butin (17.03) [272]	7,3',4',5'-tetrahydroxyflavanone (14.4)	Flavanone

Enzyme assays were run on microsome preparations of yeast transformed with the *CYP75A31 *gene. Product formation was analysed by HPLC and MS. HPLC retention times in minutes are given in parenthesis. Masses in g/mol, as determined by MS, are given in brackets.

### Gene expression

**Figure 4:**
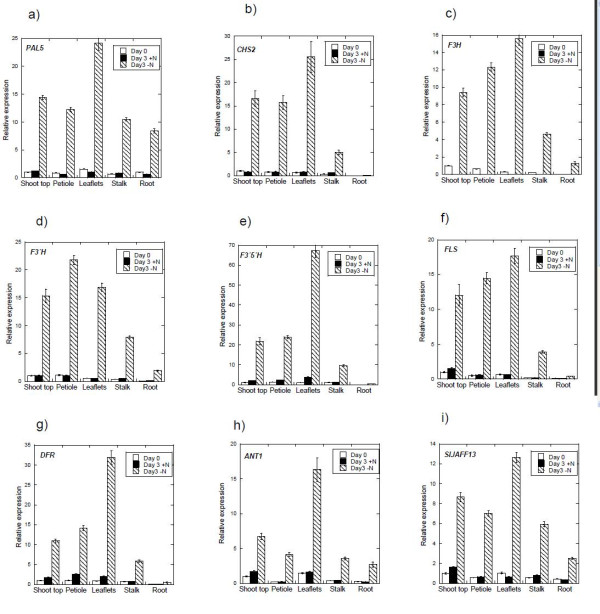
**Expression analysis by real-time PCR**. Relative expression of genes in the flavonoid pathway in various parts of the tomato plant. Tomato plants were grown for 25 days on rock-wool with complete nutrient supply under continuous light. The rock-wool was rinsed with water to remove previous nutrient solution, and plants were randomly divided in two batches. Half the plants continued with complete nutrient solution, whereas the other half received nutrient solution with no nitrogen. Samples were taken before change of nutrients (day 0) and again after three days. One biological sample was pooled from 3 different plants. Relative expression is given as a fold change related to the sample shoot top, day 0. Three analytical replicates were performed, SE is given (n = 3). Ubiqutin and elongation factor 1 α have been used as endogenous controls.

Tomato plants were grown on rock-wool with complete nutrient supply under continuous light. The rock-wool was rinsed with water to remove previous nutrient solution, and plants were randomly divided in two batches. One batch continued with complete nutrient solution, whereas the second batch received nutrient solution with no nitrogen. Samples were harvested before change of nutrients (day 0) and again after three days. Gene expression was measured by real-time PCR, using the shoot top (young tissue, e.g. shoot apex with primordia and developing leaves, including first unfolded still small leaf) on day 0 as calibrator. Relative expression of all genes is hence given as a fold change related to the shoot top sample taken on day 0. Expression of the *F3'5'H *gene, six other structural genes of the phenylpropanoid pathway and transcription factors *anthocyanin 1 *(*ANT1*) and *SlJAF13 *(which is a putative homolog to the petunia *JAF13 *gene [26]) was tested by real-time PCR. All nine genes showed a general increase in response to nitrogen deprivation (Figure 4a-i). Averaged over all parts of the plant the expression of *chalcone synthase 2 *(*CHS2*), *F3'H*, *PAL5*, *FLS*, *F3'5'H*, *DFR, SlJAF13 *and *ANT1 *on day 3 was 22.0, 19.6, 16.2, 15.7, 13.3, 8.9, 8.9 and 8.0 fold higher, respectively, in nitrogen deprived plants as compared to plants given full nutrient solution. At day 3, *flavanone 3-hydroxylase *(*F3H*) (Figure 4c) showed detectable expression only for nitrogen deprived plants, which overall was 20 fold higher than on day 0. *F3H *is the only gene with no detectable transcripts in plants receiving nitrogen on day 3; the reason for this is unknown. All of the genes, with the exception of *F3'H *(Figure 4d), showed highest expression in nitrogen depleted leaflets (from 5^th ^leaf from the hypocotyl) on day 3. For *F3'H *the highest expression was found in nitrogen depleted petioles (from 5^th ^leaf from the hypocotyl). The nitrogen effect in leaflets was especially high for *F3'5'H *(Figure 4e). *PAL5 *(Figure 4a) showed a clear increase in response to nitrogen deprivation, also in roots. *SlJAFF13 *(Figure 4i) showed a clear nitrogen effect in all plant parts tested, as did *ANT1 *(Figure 4h). Expression of *CHS2 *(Figure 4b) displayed a convincing nitrogen effect in shoot top, petiole, leaflets and stalk (of the whole plant). *DFR *(Figure 4g) was expressed in much the same way as *CHS2 *but showed a slightly higher increase in relative expression in the leaflets, and lower in the shoot top of nitrogen deprived plants. Expression of *FLS *(Figure 4f) was clearly elevated in all parts of nitrogen deprived plants while the level remained relatively stable in plants receiving nitrogen.

### Phenolic content

**Figure 5:**
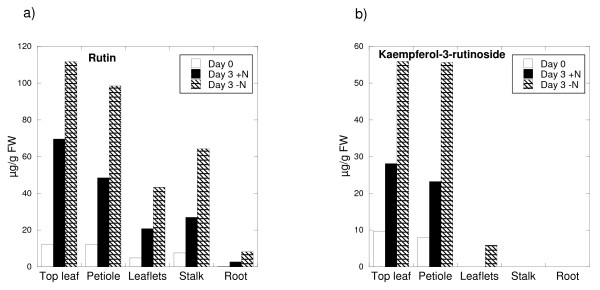
**Accumulation of flavonoids**. Accumulation of flavonoids in vegetative parts of tomato plants was determined by HPLC using standards. Tomato plants were grown for 25 days on rock-wool with complete nutrient supply under continuous light. The rock-wool was rinsed with water to remove previous nutrient solution, and plants were randomly divided in two batches. Half the plants continued with complete nutrient solution, whereas the other half received nutrient solution with no nitrogen. Samples were taken before change of nutrients (day 0) and again after three days. One biological sample was pooled from 3 different plants. Three analytical replicates were run for each sample; standard error was less than 1%. Accumulation of a) rutin and b) kaempferol-3-rutinoside is given as μg/g fresh weight (FW).

Measurements of phenolic content were conducted on the same samples as the expression analysis. Rutin was detected in all samples, except roots at day 0. In all parts of the plant the content had increased from day 0 to day 3 and was clearly higher in nitrogen deprived plants (Figure 5a). The overall content of rutin in nitrogen deprived plants on day 3 was 1.9 times higher than in nitrogen replete plants. Kaempferol-3-rutinoside was not detected in samples from stalk or root, and only in nitrogen deprived leaflets. In the shoot top and petiole there was a clear increase from day 0 to day 3, especially in nitrogen depleted plants (fig 5b). The overall content of kaempferol-3-rutinoside in nitrogen deprived plants on day 3 was 2.3 times higher than in nitrogen replete plants. Anthocyanins were not detectable in any samples under the growth conditions used.

## Discussion

When starting the in vitro enzyme assays, substrates were chosen based on previous findings on accepted substrates for F3'5'H enzymes from other plants. Substrates were also chosen based on structural similarity to these compounds. With the exception of liquiritigenin, substrates found to be metabolized by CYP75A31 were also found to be metabolized by CYP75A8, which was previously isolated from *C. roseus *[15]. The Kaltenbach group also tested a petunia F3'5'H in the *E. coli *expression system used for CYP75A8, and found that the petunia F3'5'H accepted the same substrates. Whereas the *C. roseus *F3'5'H had highest activity with apigenin, the petunia F3'5'H had highest activity with naringenin [15]. For the CYP75A31 enzyme there was a clear preference for naringenin and liquiritigenin, as these substrates were metabolised also in dilute microsome preparations. In the present study, CYP75A8 was also expressed in the same yeast (expression) system as CYP75A31. K_m _for naringenin was measured to 1.20 μM for CYP75A31, and 0.83 μM for CYP75A8. Kaltenbach et al. [15] reported an apparent K_m _of 7 μM for naringenin when expressing CYP75A8 in the *E. coli *expression system. The rate of hydroxylation performed by a F3'5'H enzyme is dependent on the reductase used in the expression system. De Vetten et al. [27] has shown that a cytochrome b_5 _is required for full activity of F3'5'H in petunia. The gene encoding a cytochrome b_5 _was inactivated by targeted transposon mutagenesis, which resulted in reduced F3'5'H activity and reduced accumulation of 5'-substituted anthocyanins, leading to an alteration in flower colour. Our expression studies utilized the Arabidopsis ATR1 reductase, whereas in the expression studies performed by Kaltenbach et al. [15], a *C. roseus *P450 reductase was used in the *E. coli *expression system. The use of different expression systems, and reductases, may explain the difference in K_m _values obtained for the *C. roseus *CYP75A8 enzyme in the two studies [28].

Liquiritigenin has to our knowledge not been shown to be metabolized by a F3'5'H enzyme previously. Liquiritigenin in plants is mostly associated with the legumes, which have a CHI capable of isomerising 6'-hydroxy- and 6'-deoxychalcones to 5-hydroxy- and 5-deoxyflavanones respectively. Joung et al. [29] reported that the tobacco CHI is able to isomerise the 6'-deoxychalcone isoliquiritigenin to the 5-deoxyflavanone, liquiritigenin, in transgenic tobacco over-expressing a *Pueraria montana *chalcone reductase gene. Tanaka et al. [20] showed that the F3'5'H from *Gentiana triflora *catalysed the hydroxylation of naringenin to eriodictyol, eriodictyol to 5, 7, 3', 4', 5'-pentahydroxyflavanone, dihydrokaempferol to dihydroquercetin, dihydroquercetin to dihydromyricetin and apigenin to luteolin when expressed in *S. cerevisiae *under the control of a glyceraldehyde-3-phosphate dehydrogenase promoter. The reaction rates and substrate preferences recorded in bacteria or yeast expression systems do not necessarily represent the actual rate or preference *in planta*. As demonstrated in this study, the tomato F3'5'H is capable of metabolizing liquiritigenin, although to our knowledge liquiritigenin has never been found in tomato plants.

Expression analysis showed that all the major genes of the flavonoid pathway tested, including *F3'5'H*, had a clear increase in expression as a result of three days of nitrogen deprivation (Figure 4). Despite what seemed to be a general up-regulation of the flavonoid pathway in this study, the growth conditions applied had not resulted in accumulation of anthocyanins at the time of sampling. At the time of sampling, the increase in gene expression was more prominent than the increase in level of rutin and kaempferol-3-rutinoside. As gene expression increases prior to accumulation of product this implies that accumulation of rutin and kaempferol-3-rutinoside had not yet reached the maximum. Similar studies (unpublished results) conducted on nitrogen deprived tomato plants have shown that also anthocyanins will appear over time. Possibly the concentrations of dihydrokaempferol and/or dihydroquercetin have to exceed a threshold level for F3'5'H to metabolise what FLS does not have capacity for. Similar studies [30] showed far higher levels of flavonol-derivatives than in the present study at the time of anthocyanin accumulation, which might indicate that FLS does not have the capacity to metabolise all the dihydrokaempferol/dihydroquercetin as the flow through the pathway escalates. The increase in transcripts of *F3'H *in all parts of the nitrogen deprived plants, indicates increased production of the F3'H enzyme, which hydroxylates dihydrokaempferol to dihydroquercetin. The action of this enzyme, (together with F3'5'H), might explain why the content of rutin is much higher than kaempferol-3-rutinoside, since they have dihydroquercetin and dihydrokaempferol as precursors respectively. It should be mentioned that although the *F3'H *tested here was a clear orthologue to the petunia *F3'H*, the tomato *F3'H *has not yet been cloned and characterised, hence its function still needs to be established. This is especially relevant considering that the F3'5'H present in tomato is also capable of catalysing the 3'-hydroxylation.

A similar study [30] showed accumulation of anthocyanins in leaves of nitrogen deprived tomato plants. In this study the nitrogen deprivation lasted a minimum of four days, and flavonoid content continued to increase from the fourth to the eighth day of nitrogen deprivation.

Consistent with the increase in rutin and kaempferol-3-rutinoside, the enzyme responsible for increasing flux into the phenylpropanoid pathway, *PAL5 *increased in expression as a response to nitrogen deprivation. The MYB-type transcription factor *ANT1*, and the putative bHLH transcription factor *SlJAF13*, also increased in all parts of nitrogen deprived plants. This is consistent with the general increase in all the flavonoid structural genes tested, and the increase in flavonoid content.

## Conclusions

The sequenced gene, *CYP75A31*, encodes a flavonoid 3'5'-hydroxylase which accepts luteolin, naringenin, eriodictyol, dihydrokaempferol, dihydroquercetin, kaempferol, quercetin and liquiritigenin as substrates. The ability to do 3'- and especially 5'-hydroxylation of intermediates in the flavonoid pathway places CYP75A31 at an important branch point in the regulation between flavonol and anthocyanin synthesis. Expression of the *CYP75A31 *gene increased in response to nitrogen deprivation, in accordance with other genes in the phenylpropanoid pathway, which is an expected response to abiotic stress in plants.

## Methods

### Plant Material

Suzanne F1 seeds were sown on rock wool and given Hoagland nutrient solution containing 15 mM NO_3_^- ^[31]. RNA and DNA used to identify coding sequence and introns of the *F3'5'H *gene was isolated from plants grown in a 12 h light/dark regimen. Expression and metabolite analysis were performed on plants grown in continuous light, and given complete Hoagland solution before shifted to a nitrogen deprived regimen where KNO_3 _was replaced by KCl and Ca(NO_3_)_2_:4H_2_O was replaced by CaCl_2_.

### Identifying the *F3'5'H *gene

RNA was isolated from leaves of the cherry tomato Suzanne F1 using the RNeasy Plant Mini Kit (Qiagen, USA). To identify the 3'end of the *F3'5'H *gene the GeneRacer™ Kit (Invitrogen, USA) was used. The gene specific left primer used for the 3' end had the sequence ACAAGGATGGGAATAGTGATGGT and was based on a *F3'5'H *sequence for *Solanum tuberosum *(accession number: AY675558). The cDNA amplified was sequenced, and a nucleotide BLAST against the GeneBank (NCBI) showed close similarity to other *F3'5'H *sequences. An EST sequence was found in the TIGR database (accession number DB723744) which was assumed to be the 5' end of the gene. Based on the obtained sequences for 3' and 5' ends, new primers covering the entire gene were made. The 3' sequence was used to make the primer 75ALerevECO (GGAATTCTCAGCAACGATAAACGTCCAAAGATAG) with an additional *Eco*RI site for the 3' end of the gene. The 5' end primer, 75ALedirBAM (GGGATCCATGGCGTTACGTATTAATGAGTTATTT), includes an additional *Bam*HI site.

cDNA for cloning was made using the SuperScript™ III First-Strand Synthesis SuperMix for qRT-PCR (Invitrogen). The ORF of *CYP75A31 *was amplified by PCR introducing *Bam*HI/*Eco*RI rectriction sites upstream of the start ATG and downstream to the stop codon TGA using Platinum^® ^*Taq *DNA Polymerase High Fidelity (Invitrogen). PCR program was as follows: 95°C for 5 min, followed by 5 cycles of 95°C for 1 min, 40°C for 1 min and 72°C for 1.5 min. Then 35 cycles of 95°C for 30 sec, 55°C for 30 sec and 72°C for 1.5 min. At the end there was an extra 5 min elongation at 72°C before cooling to 4°C. The product was ligated into a TOPO vector using the pCR^® ^8/GW/TOPO^® ^TA Cloning^® ^Kit (Invitrogen) as recommended. The ligated vector was transformed into OneShot^® ^Chemically Competent *E. coli *(Invitrogen) and grown on LB-media containing spectinomycin. Several individual colonies were picked and grown to amplify and isolate the plasmids for sequencing. The obtained sequences were subjected to a BLAST search, and were shown to display significant similarities to *F3'5'H *genes isolated from other species.

### Expression Constructs

*CYP75A31 *was cut from the TOPO vector using *Bam*HIand *Eco*RI, then ligated into the *pYeDP60 *vector [32] for expression in yeast.

### Yeast Expression and microsome preparation

The yeast strain *Saccharomyces cerevisiae *WAT11, engineered to over-express the P450 reductase isoform ATR1 from *Arabidopsis thaliana *when induced with galactose [32], was used for the expression. Transformation with the *pYeDP60 *expression construct was performed as previously described by Gietz et al. [33]. Propagation of yeast cells and preparation of microsomes was done as described by Pompon et al. [32] with some modifications. Liquid SGlu, 50 ml, was inoculated by a single colony from a SGlu plate and grown at 30°C for 48 h. The culture was then transferred to 200 ml YPGlu medium, containing 20 g/l glucose, and grown at 30°C for 24 h. The yeast cells were spun down (2000 × g, 3 min) and re-suspended in YPGal medium containing 20 g/l galactose for induction of microsomes at 16°C for 24 h. Microsomes were isolated in the following way: The yeast culture was centrifuged (2 000 × g, 10 min) and the pellet re-suspended in 50 ml TEK (100 mM KCl in 50 mM Tris-HCl with 1 mM EDTA), centrifuged at 6 100 × g for 3 min and the pellet re-suspended in 2 ml extraction buffer (20 mM β-mercaptethanol, 1% BSA and 0.6 M sorbitol in 50 mM Tris-HCl with 1 mM EDTA). Glass beads were added, and the suspension was shaken in an automatic shaker (Retsch MM200 Mixer Mill, Krackeler Scientific, USA) 4 × 2 min at a vibration frequency of 30. Between two shaking cycles the suspension was placed on ice for 3 min. Portions of 10 ml extraction buffer was added to the beads 4 times, shaken and decanted to retrieve the microsomes. Extraction buffer was centrifuged for 15 min at 6 100 × g, the supernatant was filtered, and MgCl_2 _added to a final concentration of 50 mM in order to precipitate the microsomes [34]. The suspension was placed on ice for approximately 1 h before centrifugation at 12 500 × g for 20 min. The pellet was dissolved in 1.0 to 1.5 ml TEG (30% glycerol in 50 mM Tris-HCl with 1 mM EDTA) and homogenized using a Teflon pestle. Work was carried out on ice, all buffers/solutions and centrifuge were pre-cooled to 4°C.

### CYP75A31 Enzyme assays

Several compounds were tested as potential substrates for CYP75A31. Microsomes isolated from yeast CYP75A31 transformants were incubated in 0.1 M sodium phosphate buffer, pH 7.0 containing 1.0 mM NADPH, or without NADPH (as a negative control). The assay mixture was equilibrated for 2 min at 27°C prior to starting the reaction by addition of microsomes. Concentration of substrate in the assays ranged between 20 to 100 μM. Total volume of assay was 200 μl. After 10 to 30 min the reaction was stopped by adding 75 μl of acetonitrile/concentrated HCl (99:1). Precipitated proteins were removed by a 10 min centrifugation (9300 × g); the supernatant was used directly for HPLC and MS analysis to assess product formation and substrate consumption. To validate that hydroxylations occurred due to CYP75A31 activity, assays were run with a microsome preparation made from WAT11 transformed with the *pYeDP60 *vector without any insertions.

### Real-Time PCR

Plants were sown on rock-wool and grown at 22°C for 25 days with full Hoagland nutrient solution, in continuous light (approximately 200 μmolm^-2^s^-1 ^PAR). The rock-wool was rinsed thoroughly with tap water to remove nutrients, before adding nutrient solution deprived of nitrogen (referred to as day 0). The following samples were taken from three plants and pooled to one sample (for each part of the plant): shoot top (young tissue, e.g. shoot apex with primordia and developing leaves, including first unfolded still small leaf), petiole (from the 5^th ^leaf from the hypocotyl), leaflets (from the 5^th ^leaf from the hypocotyl), stem (the whole stem of the plant) and roots (efforts were made to retrieve as much of the root as possible, but some finer parts were lost in the rock wool). The tissues were snap frozen in liquid nitrogen and stored at -80°C before ground into powder in liquid nitrogen (samples for RNA and phenolic analysis were taken from the same powder). Samples were pooled from three plants receiving nitrogen and three plants deprived of nitrogen at day three. Total RNA was isolated using RNeasy^® ^Plant Mini Kit (Qiagen). RNA was quantified by spectrophotometer and cDNA synthesised using the High Capacity cDNA Archive Kit (Applied Biosystems, USA) (concentration of RNA in the reaction tube was 10 μg mL^-1^). Real-time PCR reactions were assayed using an ABI 7300 Fast Real-Time PCR System (Applied Biosystems) with SybrGreen for detection. The reaction volume was 20 μL containing 10 μl qPCR Master Mix (PrimerDesign, UK), 0.3 μM primer (forward and reverse) and 1 μl cDNA. Standard cycling conditions (2 min at 50°C, 10 min at 95°C and 40 cycles altering between 15 s at 95°C and 1 min at 60°C) were used for product formation. Forward and reverse primers were as follows (with RTPrimerDB http://www.rtprimerdb.org identification number given in brackets when available); PAL5-F, 5'-TTTCTCCATTACAAATCAAACCA-3' and PAL5-R, 5'-TTCACTTCATCCAAATGACTCC-3', CHS2 *LOC778295 *(7794); DFR *LOC544150 *(7795); FLS-F, 5'-TAAGATTTGGCCTCCTCCTG-3' and FLS-R, 5'-ACCAAGCCCAAGTGATAAGC-3'; F3H-F, 5'-AGTGGTGAATTCGAATAGCAGTAG-3' and F3H-R, 5'-TTTCCTCCTGTACATTTCTGCAA-3'; F3'H-F, 5'-GAGGAGTTCAAGTTAATGGTGGT-3' and F3'H-R, 5'-ACTCGCTTTTCCTTGTGTTCTT-3'; ANT1 (7793); JAF13-F, 5'-AGGAGAGTTCAGGAGCTGGAG-3'; JAF13-R, 5'-GCCTTCCTTTTGTTCGGTAG-3' [30] and; F3'5'H-F, 5'-TCCCTCAACGCCACTAAATC-3' and F3'5'H-R, 5'-TTTTCCCGCTAAGGAACC-3'. Gene expression for each sample was calculated on three analytical replicates normalized using the geometric average of the reference genes ubiqutin and elongation factor 1α [35] in the qBaseplus software [36], using the shoot top harvested at day 0 as calibrator. Thus, relative quantity of any gene is given as fold change relative to day 0.

### Flavonoid standards

Naringenin, dihydroquercetin, kaempferol and quercetin were obtained from Sigma-Aldrich (USA). Liquiritigenin was obtained from Extrasynthèse (France). Luteolin, eriodictyol and dihydrokaempferol were obtained from TransMIT (Germany).

### HPLC and MS analysis

#### Analysis of enzyme substrates and products

The flavonoids were analysed on a HPLC system (LC 20AD, Shimadzu Corporation, Japan) equipped with a C18 LichroCART 125-4 column (Merck, Germany) connected to a diode array detector (SPD M20A, Shimadzu Corporation). Substrates and products separations were done using a solvent system of (A) 0.1% (v/v) acetic acid in water and (B) methanol:acetonitril (1:1). The column was equilibrated in solvent A at a flow rate of 0.9 ml/min for 5 min, and the elution was performed using a linear gradient of solvent B from 0 to 67% for 25 min, followed by 100% B for an additional 5 min. Detection was made on a wavelength range of 220-400 nm. Injection volume was 50 μl.

#### Mass spectrometric analyses

The HPLC-MS system comprised the binary solvent delivery pump (Surveyor MS, ThermoFinnigan, USA) connected to a diode array detector (Surveyor PDA plus, ThermoFinningan) and a linear ion trap mass spectrometer (LTQ-MS, ThermoFinnigan). Products separation was done as described in the above paragraph. LTQ equipped with an atmospheric pressure ionization interface operating in ESI mode. Data were processed using LCQuan software (version 2.0). Computer was controlled by Xcalibur 1.4 software. The operational parameters of the mass spectrometer were as shown below. The spray voltage was 5 kV and the temperature of the heated capillary was set at 200°C. The flow rates of sheath gas, auxiliary gas, and sweep gas were set (in arbitrary units min-1) to 50, 10, and 10, respectively. Capillary voltage was +20/-20V (positive/negative polarity), tube lens was +65/-65V (positive/negative polarity) and the front lens was +5/-5V (positive/negative polarity).

#### Characterisation of product formation

The products eriodictyol, dihydroquercetin and quercetin were identified using HPLC-standards, and MS (table 1). Triecetin, 5,7,3',4',5'-pentahydroxyflavanone, dihydromyricetin and myricetin were identified by MS (table 1). Absorbance maximum for substrates and products are given in Additional file 1. Structure for substrates and products are given in Additional file 2.

#### Analysis of flavonoids in vegetative parts of the tomato plant

Samples of approximately 100 mg were extracted in 1 ml of 1% (v/v) trifluoroacetic acid (TFA) in methanol, and analyzed by use of a liquid chromatograph (Agilent 1100-system, Agilent Technologies, Norway) supplied with a photodiode array detector. Separation was achieved on an Eclipse XDB-C8 (4.6 × 150 mm, 5 μm) column (Agilent Technologies) by use of a binary solvent system consisting of (A) 0.05% TFA in water and (B) 0.05% TFA in acetonitrile. The gradient (%B in A) was linear from 5 to 10 in 5 min, from 10 to 25 for the next 5 min, from 25 to 85 in 6 min, from 85 to 5 in 2 min, and finally recondition of the column by 5% in 2 min. The flow rate was 0.8 ml/min, 10 μl samples were injected on the column, and separation took place at 30°C. Detection was made over the interval 230-600 nm in steps of 2 nm in order to obtain full absorbance spectrum of the compounds of interest. Peak characterization was done in accordance to previous results [37,38]. Quantitative levels of the rutinosides of kaempferol and quercetin, the major flavonoids in tomato seedlings, were calculated as peak areas obtained at 370 nm compared to the responses of authentic samples (rutin and kaempferol-3-rutinoside, provided by PlantChem, Norway). All results were corrected against the exact weight of the sample. One biological sample, pooled from three individual plants, was analyzed. Three analytical replicates were done for each sample; standard error was less than 1%.

#### Phylogenetic analysis

Protein sequences of previously published F3'5'H enzymes were obtained from the NCBI home page (accession numbers are given in the phylogenetic tree, figure 3). The phylogenetic analysis was done using the default settings of ClustalX (1.83).

## List of abbreviations

4CL: 4-coumarate: CoA ligase; ANS: anthocyanidin synthase; ANT1: anthocyanin 1; bHLH: basic-helix-loop-helix; C4H: cinnamate 4-hydroxylase; CHI: chalcone isomerase; CHS2: chalcone synthase 2; DFR: dihydroflavonol 4-reductase; F3H: flavanone 3-hydroxylase; F3'H: flavonoid 3'-hydroxylase; F3'5'H: flavonoid 3'5'-hydroxylase; FLS: flavonol synthase; PAL5: phenylalanine ammonia-lyase 5; TFA: trifluoroacetic acid; UFGT: UDP glucose flavonoid 3-O-glucosyl transferase.

## Authors' contributions

KMO performed the cloning and expression studies, HPLC analysis on enzyme assays, real-time PCR analysis, and drafted the manuscript. AH provided guidance and help in cloning and expression studies. HJ did expression studies and measured K_m_. RS performed HPLC analysis on enzyme assays, and measured phenolic content in tomato plant samples. RL gave guidance in HPLC, and did MS analysis. FB defined the project presented and provided general advice. CL provided general advice, especially on writing of article. All authors read and approved the final manuscript.

## Supplementary Material

Additional file 1 **Absorption maximum for substrates and products**. HPLC absorption maximum for substrates and products used. Click here for file

Additional file 2 **Structures of substrates and products**. Structures for substrates and products. Click here for file
