# Supplementary material for: Identification and characterisation of CYP75A31, a new flavonoid 3'5'-hydroxylase, isolated from Solanum lycopersicum
Source: BMC Plant Biol. 2010 Feb 3;10:21. doi: 10.1186/1471-2229-10-21 (PMC2825239; doi:10.1186/1471-2229-10-21)
Supplement: Additional file 1 — Absorption maximum for substrates and products. HPLC absorption maximum for substrates and products used. [file 1471-2229-10-21-S1.PDF]

Absorption maximum for substrates and products.

Lambda max (nm) is given in parenthesis. Sh means shoulder.

**Substrate**	**Product of 3’- hydroxylation**	**Product of 5’- hydroxylation**
Luteolin (349)	-	Tricetin (356)
Naringenin (288, 325 sh)	Eriodictyol (288, 325 sh)	5,7,3',4',5'-pentahydroxyflavanone (282, 325 sh)
Eriodictyol (288, 325 sh)	-	5,7,3',4',5'-pentahydroxyflavanone (282, 325 sh)
Dihydrokaempferol (289, 325 sh)	Dihydroquercetin (288, 330 sh)	Dihydromyricetin (289, 330 sh)
Dihydroquercetin (288, 330 sh)	-	Dihydromyricetin (289, 330 sh)
Kaempferol (365)	Quercetin (370)	Myricetin (370)
Quercetin (370)	-	Myricetin (370)
Liquiritigenin (275, 312 sh)	Butin (278, 312 sh)	7,3',4',5'-tetrahydroxyflavanone (274, 313 sh)
