# Supplementary material for: Identification and characterisation of CYP75A31, a new flavonoid 3'5'-hydroxylase, isolated from Solanum lycopersicum
Source: BMC Plant Biol. 2010 Feb 3;10:21. doi: 10.1186/1471-2229-10-21 (PMC2825239; doi:10.1186/1471-2229-10-21)
Supplement: Additional file 2 — Structures of substrates and products. Structures for substrates and products. [file 1471-2229-10-21-S2.PDF]

Structures of substrates and products

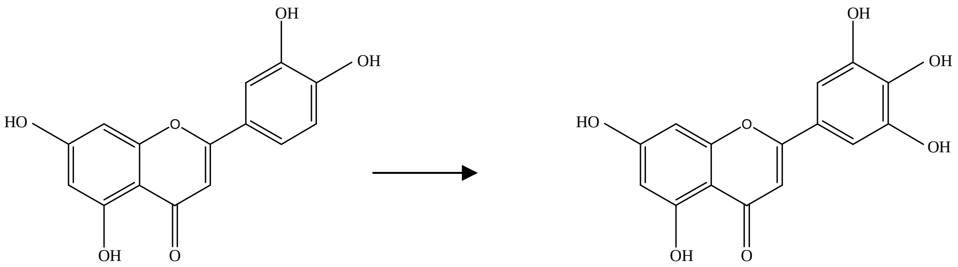

Luteolin
(5,7,3',4'-tetrahydroxyflavone)

Tricetin
(5,7,3',4',5'-pentahydroxyflavone)

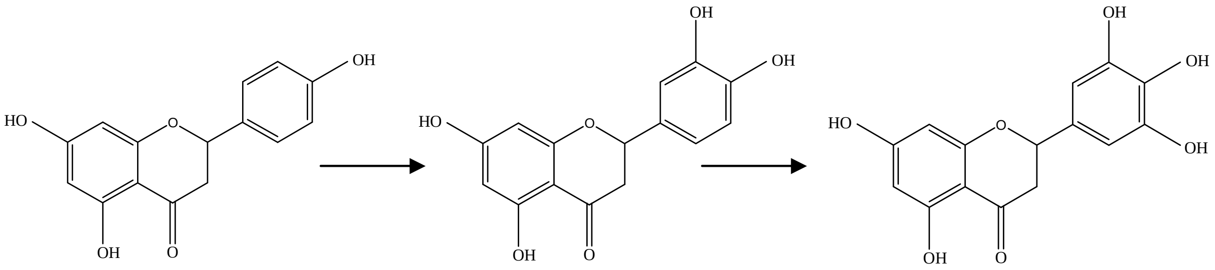

Naringenin
(5,7,4'-trihydroxyflavanone)

Eriodictyol
(5,7,3',4'-tetrahydroxyflavanone)

5,7,3',4',5'-pentahydroxyflavanone

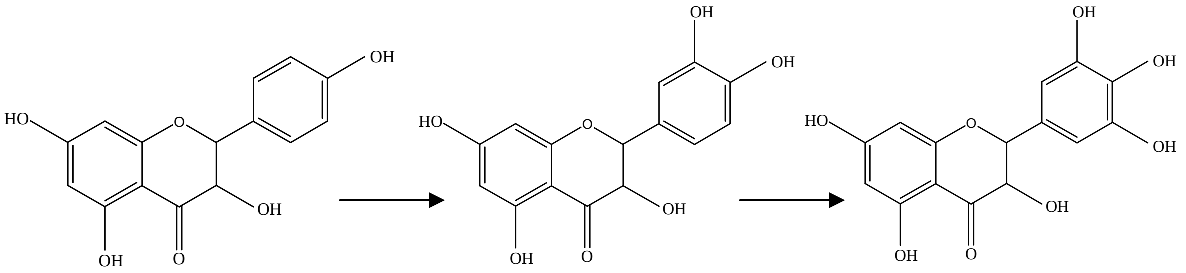

Dihydrokaempferol
(3,5,7,4'-tetrahydroxyflavanone)

Dihydroquercetin
(3,5,7,3',4'-pentahydroxyflavanone)

Dihydromyricetin
(3,5,7,3',4',5'-hexahydroxyflavanone)

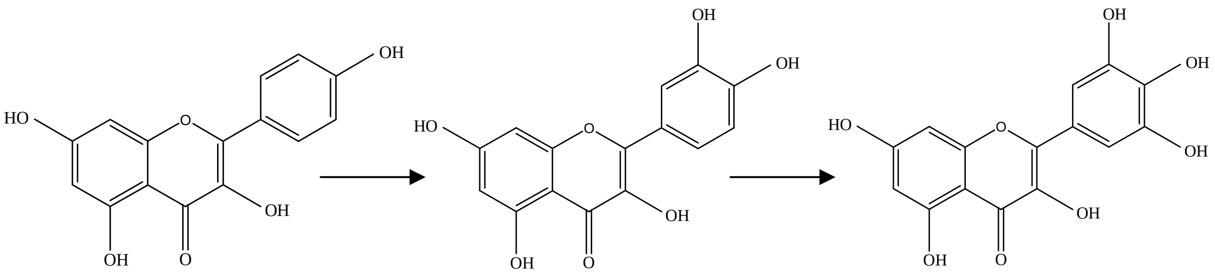

Kaempferol
(3,5,7,4'-tetrahydroxyflavone)

Quercetin
(3,5,7,3',4'-pentahydroxyflavone)

Myricetin
(3,5,7,3',4',5'-hexahydroxyflavone)

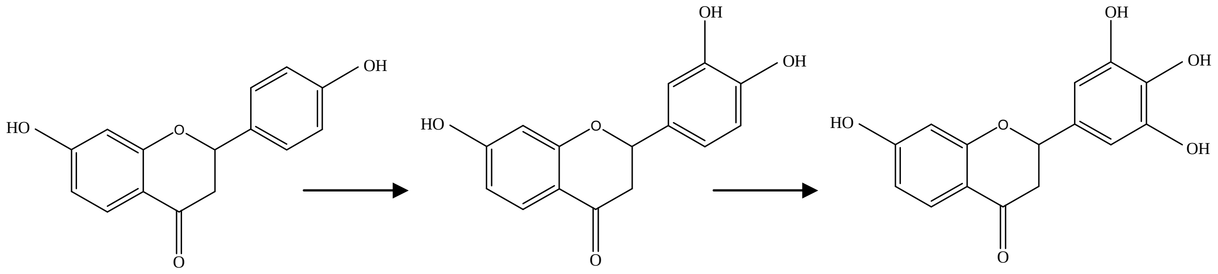

Liquiritigenin
(7,4'-dihydroxyflavanone)

Butin
(7,3',4'-trihydroxyflavanone)

7,3',4',5'-tetrahydroxyflavanone

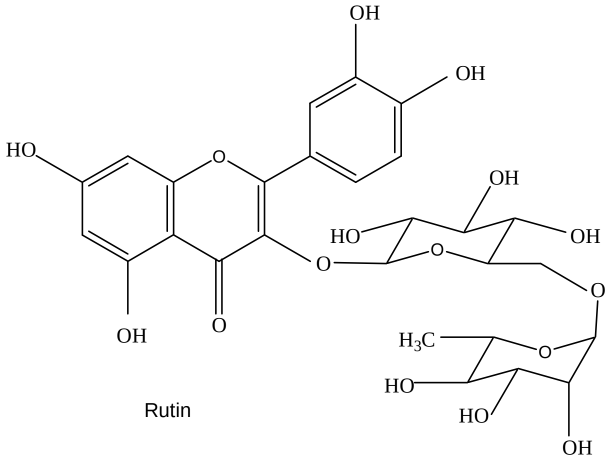

Rutin

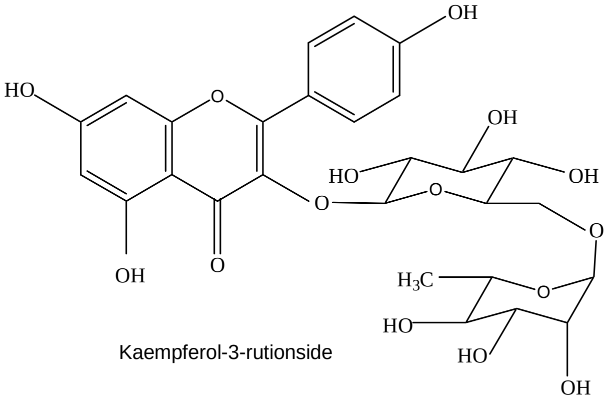

Kaempferol-3-rutionside
